# Construction of reusable fluorescent assembled 3D-printed hydrogen-based models to simulate minimally invasive resection of complex liver cancer

**DOI:** 10.1371/journal.pone.0316199

**Published:** 2024-12-27

**Authors:** Wenli Cao, Xiaofeng Pan, Liming Jin, Jie Liu, Jie Cao, Lei Jin, Fangqiang Wei

**Affiliations:** 1 Department of General Surgery, Cancer center, Division of Hepatobiliary and Pancreatic Surgery, Zhejiang Provincial People’s Hospital, Affiliated People’s Hospital, Hangzhou Medical College, Hangzhou, Zhejiang Province, China; 2 Department of Public Health, Hangzhou Medical College, Hangzhou, Zhejiang Province, China; 3 Department of General Surgery, Yunhe County People’s Hospital, Yunhe, Zhejiang Province, China; 4 Ningbo Chuangdao 3D Medical Technology Co., Ltd., Ningbo, Zhejiang Province, China; 5 Second Clinical Medical College, Zhejiang Chinese Medical University, Hangzhou, Zhejiang Province, China; University of Marburg: Philipps-Universitat Marburg, GERMANY

## Abstract

Complex liver cancer is often difficult to expose or dissect, and the surgery is often challenging. 3D-printed models may realistically present 3D anatomical structure, which has certain value in planning and training of liver surgery. However, the existing 3D-printed models are all monolithic models, which are difficult to reuse and limited in clinical application. It is also rare to carry fluorescence to accurately present tumor lesions. Here we report reusable fluorescent assembled 3D-printed models to mimic minimally invasive resection of complex liver cancer. Based on the models, multiple copies of liver lesion structure assembled accessories can be printed for the same patient or different patients, ensuring the quantity and quality of simulated surgical training, and greatly reducing the cost of simulated surgical training. The addition of fluorescence is helpful in accurately presenting tumor lesions. The reusable fluorescent assembled 3D-printed models may mimic minimally invasive resection of complex liver cancer, demonstrating potential value in simulated surgery.

## 1. Introduction

In recent years, 3D printing technology has been widely used in various fields of medicine including complex liver cancer resection [1, 2]. 3D-printed liver models can visualize liver anatomical structure and complex spatial structure of tumor, plan preoperative surgical navigation, improve anatomy understanding, which may be helpful for facilitating liver resection and medical training [2, 3]. However, complex processes involving image segmentation and model creation often require extensive labor and a steep learning curve, taking up several days [4–6]. And manufacturing of the 3D-printed models, along with the essential software and materials, can be prohibitively expensive [7–10]. This makes the use of 3D-printed models limited in surgical training. In addition, most 3D-printed liver models can only be used for clinical medicine education [11], preoperative anatomic planning [12], 3D visualization of liver diseases [13], and cannot be used by surgeons to conduct simulated surgery. Moreover, although some of the existing reported 3D-printed training models are 1:1 replicas of the patient’s liver [14], since these models are customized for the patient and are specific, they can only be used once.

Specifically, when performing repeated liver resection, trainees need to replace the whole liver because the existing 3D-printed models in surgical training cannot be reused [14]. Moreover, currently, there are rarely any liver models made of hydrogel that allow for procedures such as electrocautery, which simulates real surgical operations. Additionally, the existing 3D-printed models are also rare to carry fluorescence to accurately present tumor lesions. There are also no reusable models available where 3D-printed hydrogen-based models can be assembled to simulate minimally invasive resection of complex liver cancer. Collectively, the existing 3D-printed models are all monolithic, featuring high costs, long production times, limited reusability, scant use of hydrogel materials, lack of fluorescence, restricted clinical applications, and difficulty in achieving effective improvements in repetitive training for key surgical steps.

It’s noteworthy that the advent of artificial intelligence (AI) and virtual reality (VR) in recent years has revolutionized laparoscopic training, making it increasingly convenient and effective [15–19]. AI and VR may provide highly detailed and realistic simulations of laparoscopic procedures. This enhanced visualization may help trainees develop a deeper understanding of surgical techniques and anatomy. VR-simulators may provide an opportunity for endless repetition and refinement of skills, which is crucial for developing proficiency in surgical techniques and has been increasing used in practicing laparoscopic procedures [15–19]. However, participants often cannot physically touch the simulated objects, resulting in a lack of tangibility. In surgical training, tactile sense is extremely important as it helps doctors understand tissue texture, judge the cutting force and depth of surgical instruments, and so on. In contrast, 3D-printed realistic laparoscopic training models allow doctors to directly manipulate the models, providing a more authentic tactile and operational experience. Besides, the development and maintenance of VR simulation training systems require advanced technical support, which typically translates into higher costs that may be prohibitive, particularly for those smaller, non-academic institutions that train few trainees [15, 16]. Additionally, system configuration and updates may also require additional investments. While 3D-printed realistic models also entail certain equipment and material costs, compared to complex virtual reality systems, their costs may be more manageable.

Here we report reusable fluorescent assembled 3D-printed hydrogen-based models that may effectively address the issue of repeated training for minimally invasive surgery in complex liver cancer cases and enhances training efficiency. Based on the models, multiple copies of liver lesion structure assembled accessories can be printed for the same patient or different patients, ensuring the quantity and quality of simulated surgical training, and greatly reducing the cost of simulated surgical training. The addition of fluorescence is also helpful in accurately presenting tumor lesions. The purpose of this paper is to demonstrate the development process of a workflow for modeling and utilizing models in surgical training. It aims to provide a practical and feasible repeated training model for minimally invasive resection of complex liver cancer, thereby reducing costs and improving training efficiency.

## 2. Methods

### 2.1. Patient cohort and data collection

From January 1^st^ 2023 to March 31^st^ 2024, two patients with complex liver cancer who were treated at our center were enrolled in the study and related clinical and imaging data were retrospectively reviewed. Data were fully accessed for research purposes at April 30th 2024 when human research ethics approval was obtained. This study was conducted in accordance with the Declaration of Helsinki, and the study protocol was approved (Approval NO.: ZJPPHEC 2024O(093)) by the Ethics Committee of Zhejiang Provincial People’s Hospital. Due to the retrospective nature of this study, patient consent for inclusion was waived by the Ethics Committee of Zhejiang Provincial People’s Hospital.

### 2.2. Model development

#### 2.2.1. Design of silicone-based 3D models of liver immobilized block

**Fig 1:**
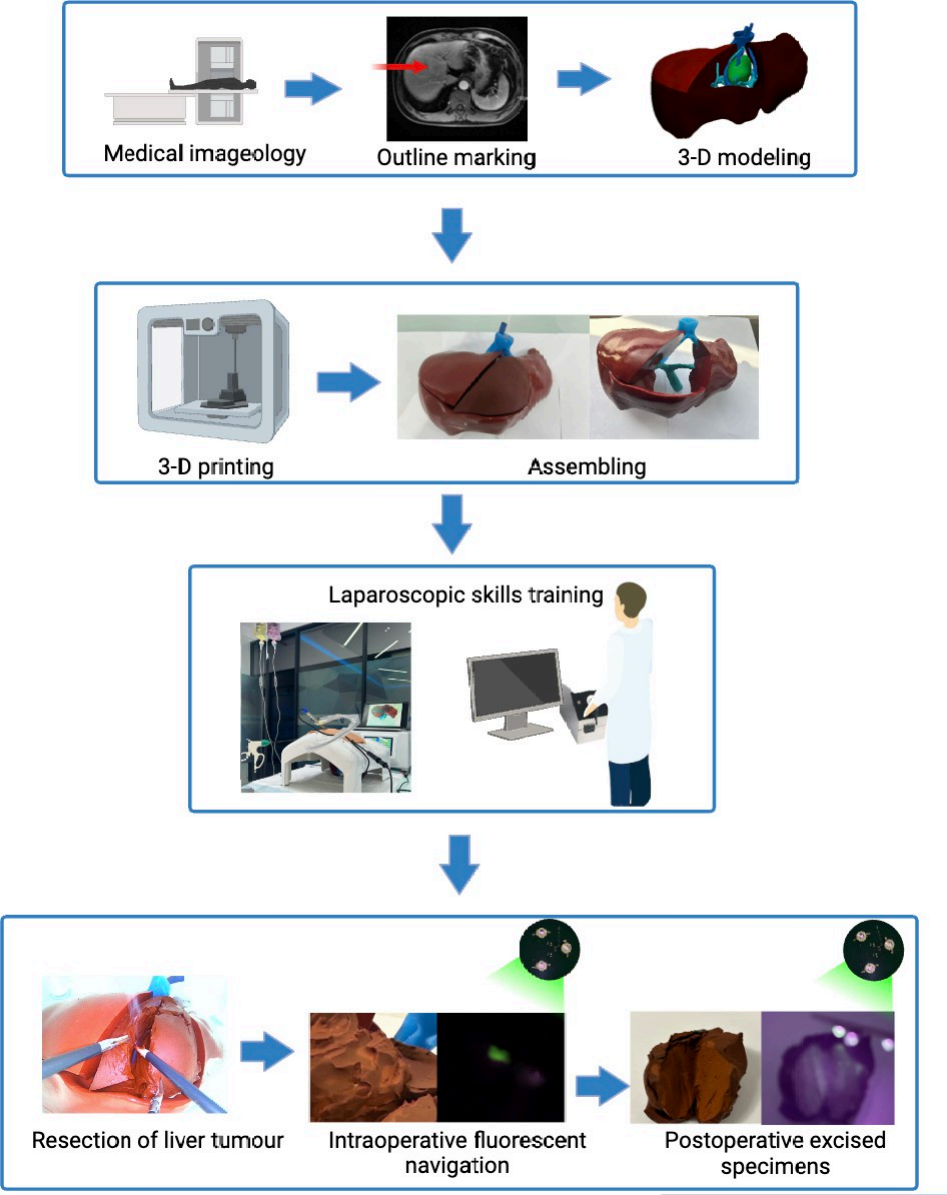
Workflow of simulated surgery using reusable fluorescent assembled 3D-printed hydrogen-based models.

**Fig 2:**
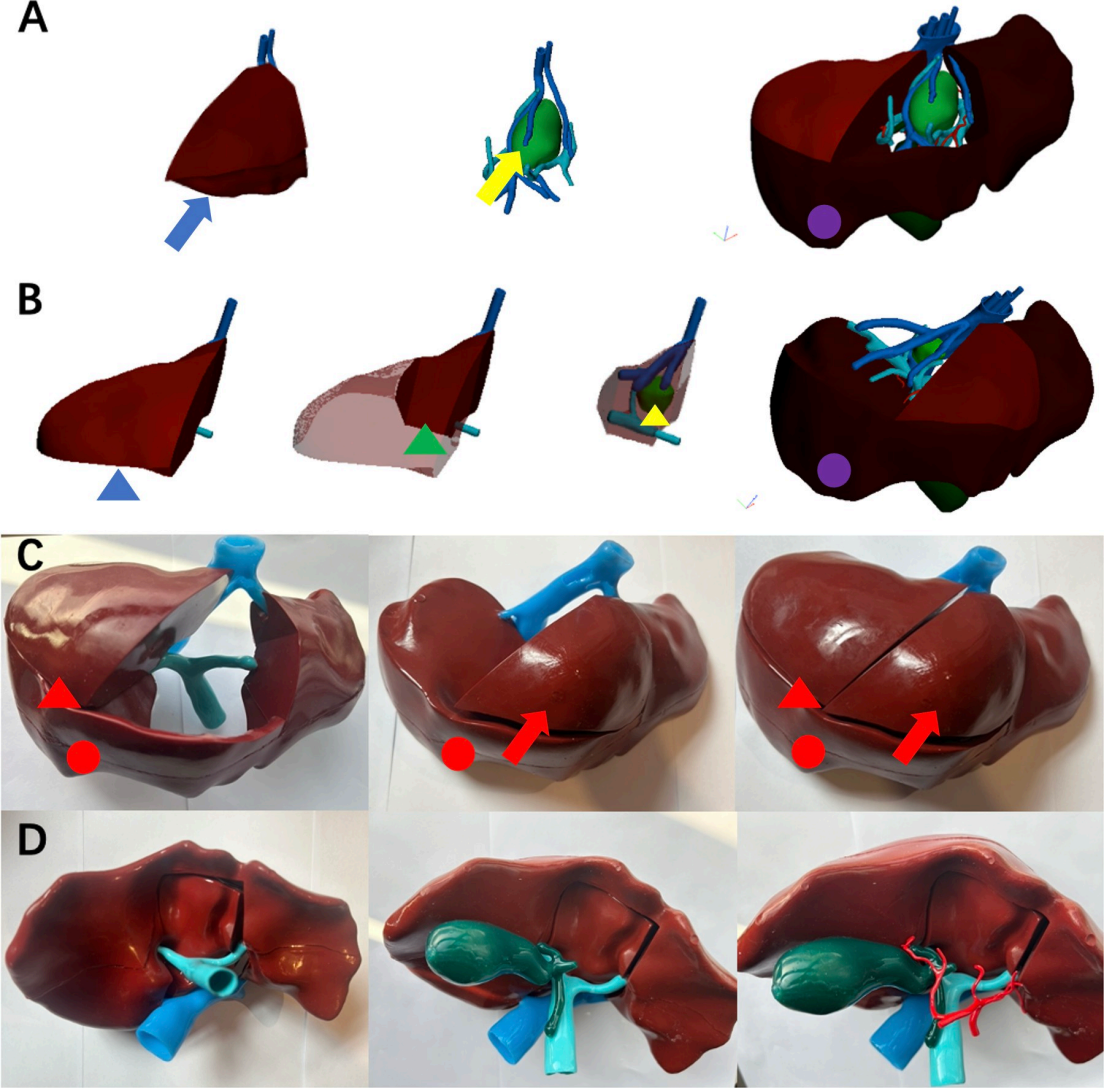
Design and creation of 3D liver models. A: The electronic design of the middle liver assembled block (marked by the blue arrow) with a liver tumor located at segment 4a (marked by the yellow arrow). Additionally, an immobilized liver block has been designed (marked by the purple circle). B: The electronic design of the right liver assembled block (marked by the blue triangle) incorporates a smaller, movable assembled block (marked by the green triangle). Within that smaller block, a liver tumor is located at segment 8 (marked by the yellow triangle). Additionally, an immobilized liver block has been designed (marked by the purple circle). C: Creation of real 3D silicone-based models for the liver immobilization block (marked by the red circle), with two movable segments (marked by the red triangle and arrow) of the posterior superior region of the liver, allowing for future replacement with hydrogel models. D: Addition of auxiliary structures such as gallbladder, hepatic artery to make the model more complete.

**Fig 3:**
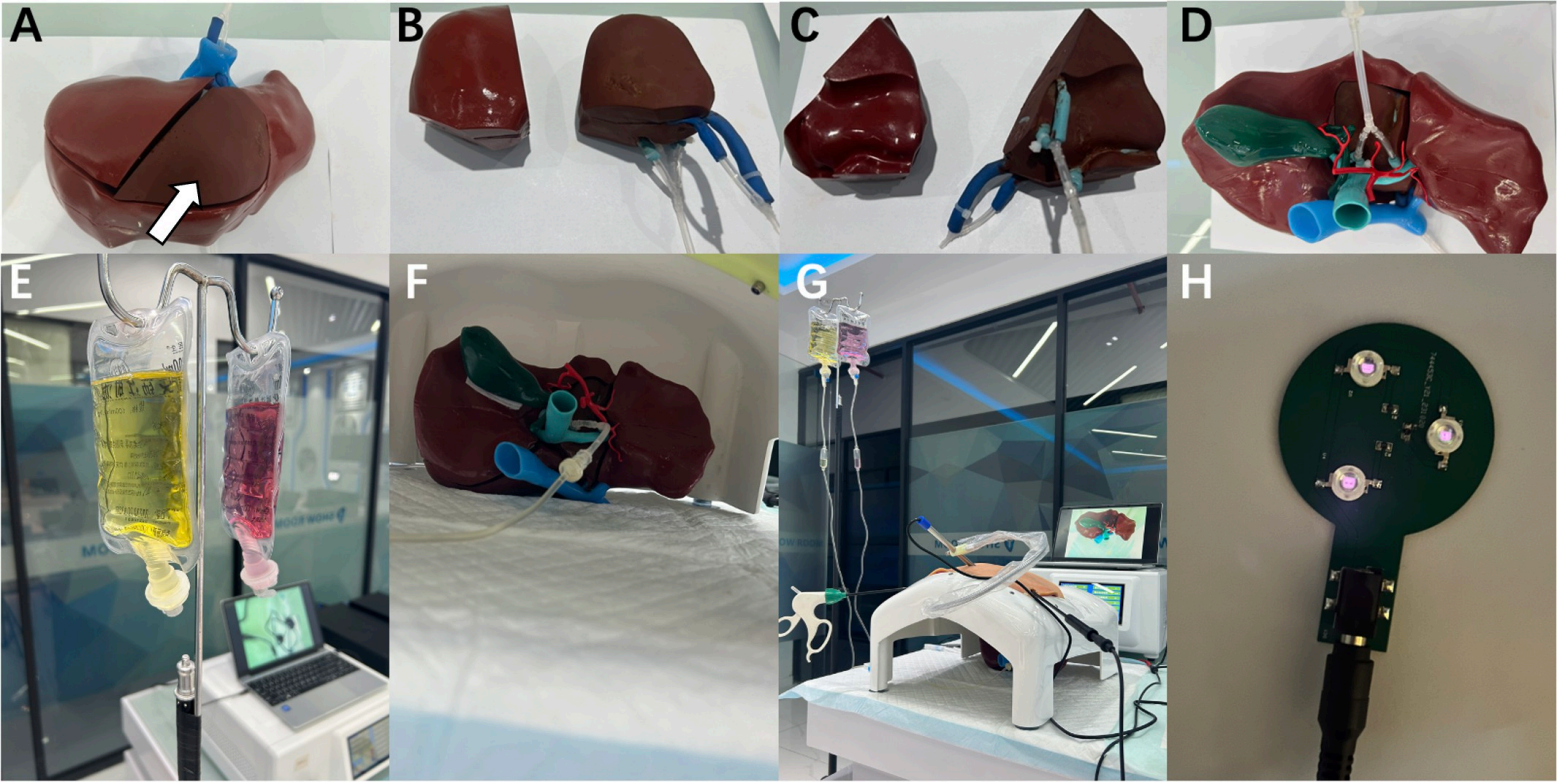
Preparation of models and simulated surgery. A,D: The hydrogel-based assembled block (marked by the white arrow) is put into the reserved groove in the silicone-based immobilized block. B,C: The silicone-based assembled block, and the hydrogel-based assembled block with the hepatic vein and portal vein branches connecting to small tubes. E: The infusion bags carrying different stained saline to simulate the blood. F: The models were placed in an abdominal cavity. G: The hepatic vein and portal vein branches in the hydrogel-based 3D models of liver assembled block are connected with infusion bags containing simulated blood to simulate blood flow. And the models were placed in an abdominal cavity simulator with a camera connected to the display screen to simulate the surgical process. H: Homemade infrared light for detection of fluorescence.

According to the shortcomings and problems of existing 3D models, we attempted to design novel 3D-printed liver models that can be reused, assembled, electrocoagulable, and carry fluorescence (Fig 1). First, we designed a silicone-based liver immobilized block. The Digital Imaging and Communication in Medicine (DICOM) files were acquired from magnetic resonance imaging (MRI) scans of a patient (patient A) with liver cancer lesions at segment 4a (Fig 2A). The DICOM data of this patient serves as the foundational data for modeling, encompassing not only the parts that can serve as the skeleton (Fig 2A, marked by the purple circle) but also a movable part (Fig 2A, marked by the blue arrow) containing the tumor (Fig 2A, marked by the yellow arrow). The E-3D Digital Medical Modeling and Design System V19.12 (Digital Health and Virtual Reality Research Center, Central South University, China) was utilized for hepatic segmentation, 3D reconstruction, design, and extraction of stereolithography (STL) format data. Next, the open source slicing software Cura 4.4.1 (Ulitmaker, USA) was used for slicing for 3D printing. Finally, the liver immobilized block was printed using silicone gel, except that two parts (Fig 2A and 2B, marked by the blue arrow and blue triangle, respectively) of the posterior superior segments of liver which could be moved for future replacement with a hydrogel model (Figs 2 and 3). The specific details regarding the 3D-printed models are as follows: these 3D-printed silicone models were produced using a dual-extrusion silicone 3D printer (Silplot-S400, Ningbo Chuangdao 3D Medical Technology Co., Ltd., China). The silicone material used in this study has a tensile strength of 2.65 MPa and an elongation of 158%.

#### 2.2.2. Design of hydrogel-based 3D models of liver assembled block with fluorescence

Next, we designed hydrogel-based 3D models of liver assembled block with fluorescence. We used the abovementioned liver immobilized block 3D models as a universal model (Fig 2C, marked by the red circle). The assembled blocks were designed according to the location of complex tumors in different liver segments such as segment 4a, segment 7, segment 8, etc. The DICOM files were derived from MRI scans of two patients (patients A and B) with liver cancer lesions, one patient with liver cancer lesion located in the liver segment 4a (Fig 2A, marked by the yellow arrow), and the other patient with liver cancer lesion located at liver segment 8 (Fig 2B, marked by the yellow triangle). This time, two moving blocks were designed according to patients A and B, and structures such as the mass and the surrounding portal vein and hepatic vein were designed within the moving blocks (Fig 2). The E-3D Digital Medical Modeling and Design System V19.12 (Digital Health and Virtual Reality Research Center, Central South University, China) was used for 3D reconstruction, design, and extraction of STL format data, and the open source slicing software Cura 4.4.1 (Ulitmaker, USA) was used for slicing for 3D printing. The SL600 laser rapid forming 3D printer (SL600, Suzhou Zhongrui Zhichuang 3D Technology Co., LTD., China) was used to print the mold of the assembled block, and the inside of the printed mold is filled with hydrogel material to form. The mold is made of photosensitive resin material (ZR680, Suzhou Zhongrui Zhichuang 3D Technology Co., LTD., China), with bending strength of 66–73 MPa and elongation at break of 10–15%. The tensile strength of the hydrogel material used in the study is 1.09 MPa and the elongation is 333%. The hydrogel model (Fig 3A, marked by the white arrow) is designed based on the varying locations of tumors and the requirements of training. Regarding to the fluorescence, we added a certain concentration of 70% of infrared fluorescent powder (purchased from Shenzhen Oriental Color Changing Technology Co., LTD., China) to the hydrogel material to create a 3D-printed fluorescent tumor model. This infrared fluorescent powder does not show color in sunlight and daylight, and displays dazzling light under near-infrared light irradiation of 950∼1000nm (peak 980nm), so that the invisible infrared band beam is converted into visible green light, and can be recorded by the Apple phone camera (Fig 1). Fluorescent tumors are hidden in the hydrogel model. After liver resection, the tumor boundary can be clearly seen by infrared excitation). Regarding the hydrogel models, including the liver block, tumor, portal vein, and hepatic vein can be cauterized by electrocoagulation. Once we finished modelling, auxiliary structures can be printed and added, such as gallbladder, hepatic artery, etc., to make the model more complete (Fig 2D).

## 3. Results

### 3.1. Workflow of simulated surgery

Based on the abovementioned work, we propose a workflow (Fig 1) based on the reusable fluorescent assembled 3D-printed hydrogen-based models for simulation of complex liver cancer resection.

**Fig 4:**
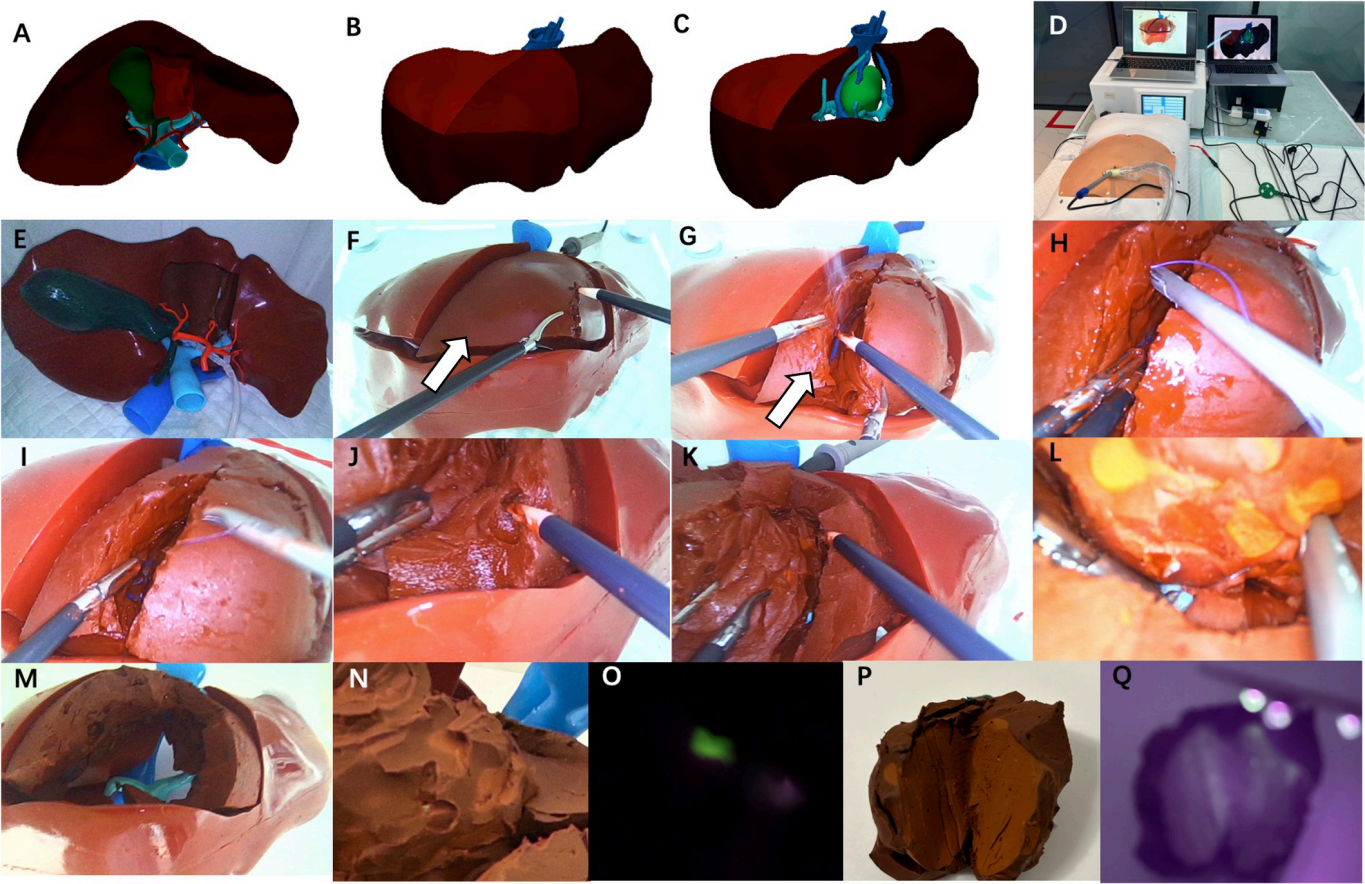
The models were used to simulate surgery for resection of liver tumor located at segment 4a. A-C: Electronic design of 3D image of the liver with liver tumor lesion at segment 4a. D,E: Models prepared well for simulated laparoscopic surgery. F,G: The hydrogel-based block (marked by the white arrow) was treated by electrocoagulation. H,I: Laparoscopic suture of the hepatic vein branch. J,K: Laparoscopic liver resection was almost completed gradually to reveal the liver tumor. L: Laparoscopic suture of the portal vein branch. M: The wound after mass removal. N,O: Intraoperative fluorescence navigation to show the tumor boundary. P,Q: After the tumor is removed, fluorescence irradiation can stain the entire tumor and its boundaries.

**Fig 5:**
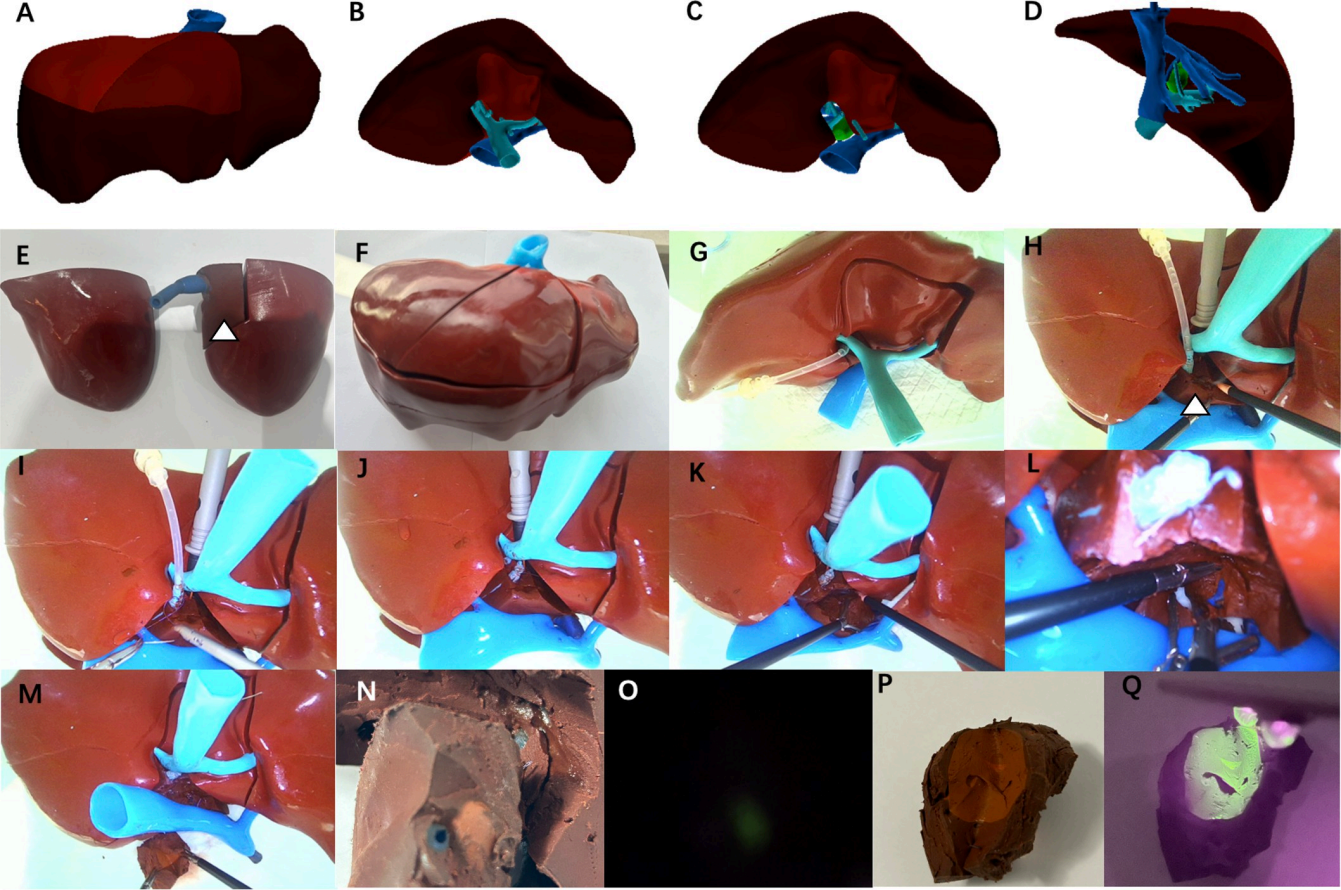
The models were used to simulate surgery for resection of liver tumor located at segment 8. A-D: Electronic design of 3D image of the liver with liver tumor lesion at segment 8. E: The whole silicone-based assembled block, and the hydrogel-based assembled block (marked by the white triangle) is embedded in the silicone-based assembled block. F,G: Models prepared well for simulated laparoscopic surgery. H: The hydrogel-based block(marked by the white triangle) was treated by electrocoagulation. I,J: Laparoscopic suture of the portal vein branch. K: Laparoscopic liver resection was almost completed gradually to reveal the liver tumor. L: Laparoscopic suture of the hepatic vein branch. M: The mass was completely removed. N,O: Intraoperative fluorescence navigation to show the tumor boundary. P,Q: After the tumor is removed, fluorescence irradiation can stain the entire tumor and its boundaries.

1. Design and creation of silicone-based 3D models of liver immobilized block. (serve as a general model for future reusable training) (Fig 2).
2. Design and creation of hydrogel-based 3D models of liver assembled block with fluorescence (create different assembled blocks according to the patients’ lesion, and create multiple same blocks for repetitive training) (Fig 3).
3. Model preparation. Put the assembled block into the reserved groove in the immobilized block, and glue the gap with tape paper (Fig 3).
4. Blood flow simulation. The hepatic vein and portal vein branches in the assembled blocks are connected with infusion bags containing simulated blood to simulate blood flow (Fig 3).
5. Simulated surgery. The models were placed in an abdominal cavity (Fig 3), and the models allow for electrical coagulation, cutting, suture and other operations (Figs 4 and 5).
6. Fluorescence display. During and after liver resection, the tumor and its boundary can be clearly seen by infrared excitation (Figs 4 and 5).

The workflow of simulated surgery include several steps. First, design a general silicone-based 3D model using DICOM data. This model serves as the frame, remains unchanged, and is used for repeated training (Fig 2). Second, design the assembled block from liver cancer patients’ DICOM data. Through software design, the authentic structure of tumor and related portal vein and hepatic vein are presented, and adjusted to the assembled block that can match the immobilized block (Fig 3). At the same time, a certain concentration of infrared fluorescent powder was added to the tumor lesions. Third, put the assembled block into the reserved groove in the immobilized block, and glue the gap with tape paper (Fig 3). The whole model is ready for the next step of training. It is worth noting that the assembled block needs to be kept moist for better electrocoagulation operation. Fourth, to better simulate the surgical scenario, we will use the infusion bag to carry different stained saline to simulate the blood flow. The hepatic vein and portal vein branches in the assembled block are connected to the infusion bag through the tube. During simulated surgery, gravity drive is used to simulate blood flow (Fig 3). Fifth, the models were placed in an abdominal cavity simulator with a camera connected to the display screen to simulate the surgical process, and the models allow for electrical coagulation, cutting, suture and other operations (Figs 3–5). Last, during and after liver resection, the tumor and its boundary can be clearly seen by infrared excitation, which can be recorded by the Apple phone camera (Figs 4 and 5).

### 3.2. Features of the models

First of all, based on 3D printing technology, we can simulate the lesions and surgical processes of complex liver cancer. When placed in an abdominal cavity simulator, our models can perfectly simulate laparoscopic complex liver cancer resection. This provides a very good platform for the surgical training of surgeons, and may even provide a possibility for future preoperative simulated surgery. Second, models can be reused. The silicone immobilized block can be used repeatedly as a universal model. We can design different hydrogel assembled blocks for different complex liver cancer lesions and adapt them to be assembled on silicone blocks. And multiple hydrogel assembled blocks can be printed for repeated training. Therefore, since only one hydrogel assembled block need to be printed each time, the single training cost of the model will be greatly reduced and the production efficiency improved. Third, the models can be electrically coagulated. The tumor, portal vein and hepatic vein in hydrogel assembled blocks can be treated by electrocoagulation. Models can also be cut and sutured. This can realistically simulate surgery of complex liver cancer. Fourth, our models incorporate fluorescent color rendering. During and after liver resection, tumor staining and tumor boundaries can be seen by fluorescence irradiation. This allows training to ensure negative margins during liver resection. Fifth, models can simulate blood flow by connecting the infusion bag, which in turn simulates bleeding during liver resection. This further makes simulated surgery more realistic.

To the best of our knowledge, this is the first reusable, electrocoagulable, assembled, fluorescent 3D-printed models to simulate minimally invasive resection of complex liver cancer.

Two hepatobiliary surgical experts from our institution commented that through these models, it may be possible to conduct training for laparoscopic resection of complex liver cancer, including tumor localization training, surgical planning training, training to ensure negative surgical margins, training for identification, protection, and transection of portal and hepatic veins, as well as training in electrocoagulation, cutting, and suturing and other procedures. This helps the surgeon master key steps in the dissection of liver cancer by repeated training through hydrogel blocks.

## 4. Discussion

In recent years, substantial progress has been made in the development of preoperative surgical planning and simulation training for complex surgeries based on 3D printing technology, which enables the production of intuitive, precise, and customized anatomical 3D models, providing valuable assistance in the field of precision medicine, particularly in addressing the intricacies of complex liver surgery, aiding surgeons in effectively navigating the complexity of liver anatomy [20]. Although more and more 3D-printed models are used for preoperative navigation and medical teaching [11–13], relatively few 3D-printed models are used for surgical training.

In conventional surgical training, cadaveric, animal, and isolated organ models are commonly used as wet laboratory models [21, 22]. However, due to concerns regarding high costs, social pressures, and biosafety issues, there is a growing need for more suitable alternatives to wet laboratory models [23, 24]. Indeed, these methods have shown a variety of shortcomings. For instance, animal and in vitro models do not accurately mimic the human body, and their uses always raises ethical issues [25, 26]. Over the past few years, VR models have made significant advancements [15–19, 26, 27]. Although they may serve as an alternative to 3D-printed models, they still lack the ability to provide physical and tactile interaction, leading to relatively impractical training experiences.

Recently, a self-healing 3D-printed model has made a breakthrough in surgical navigation and training [28]. This innovative model allows surgeons to practice resecting liver tumors repeatedly on the same lesion based on the highly efficient self-healing performance, ultimately improving the safety of liver surgery by ensuring optimal surgical margins and reducing the incidence of unforeseen injuries. Although these 3D-printed models can be repeatedly trained according to a patient’s lesions, these models are tailored to a patient’s liver and therefore cannot support training for simulation of liver lesions in different patients. Additionally, these models are still monolithic models that require a complete 3D-printing workflow for each patient, which is expensive and time-consuming, making it challenging to effectively improve training of key surgical steps.

Innovatively, we have created reusable fluorescent assembled 3D-printed hydrogen-based models, which may effectively solve the problem of repeated surgical training of crucial surgical steps in the minimally invasive treatment of complex liver cancer, and through the concept of assembled blocks, reusability, saving printing time and the cost. The silicone-based liver immobilized block design, complemented by the hydrogel-based assembled block, facilitates repetitive resection training of complex liver cancer through the replacement of the assembled block. Meanwhile, these models allow surgeons to understand in advance the location of tumor lesions, their size, the distribution of surrounding blood vessels, and other relevant anatomical structures. Specifically, in the current study, it can be seen that the lesion of patient A is located in liver segment 4a, close to the umbilical fissure vein and the middle hepatic vein (two branches) (Fig 2A), providing intuitive navigation for resection. For patient B, the lesion is located at liver segment 8, adjacent to the inferior vena cava and right hepatic vein (Fig 2B), making the complex tumor localization more intuitive and simplified.

Currently, the cost of 3D-printed liver models is high. Madurska et al. [7] developed a patient-specific 3D-printed liver model with the cost between $500–600; Huber et al. [8] reported that the costs for 3D printing ranged from $1600 to $2200 per case. According to Prashanth et al. [9], the total cost per 3D-printed model can be over $2,500. Igami et al. [10] chose to create a 3D-printed liver model at a 70% scale. Model of this size is easy to operate with one hand. And the cost is about half of the entire model. In order to reduce training costs, our models are designed to be assembled and reused. Specifically, we can produce multiple copies of assembled blocks with liver cancer lesions for the same patient, facilitating preoperative planning and ensuring the quantity and quality of simulated surgical training. Moreover, for different patients, we can replace the assembled blocks and print various types of assembled blocks with liver cancer lesions, greatly reducing the costs associated with preoperative planning and simulated surgical training. This approach also improves efficiency, as it only requires printing the assembled block.

To achieve accurate and minimally invasive treatment of complex liver cancer, it is necessary to completely resect the cancerous lesion. It has been reported that intraoperative indocyanine green (ICG) fluorescence navigation can accurately locate liver cancer lesions, help improve anatomical liver resection rate and R0 resection rate, increase resection margin distance, and ultimately improve long-term prognosis [29]. ICG is a commonly used fluorescent dye as an innovative liver surgical imaging technique that helps improve the visualization of anatomical structures through real-time liver mapping [30]. When the tumor is located in a challenging location, such as the right posterior lobe, residual ICG within the lesion may fluoresce under near-infrared light, helping surgeons quickly identify the lesion [31]. Since fluorescence navigation may improve surgical resection rate and avoid positive surgical margins, our goal is to incorporate this characteristic into surgical training. Similarly, our 3D-printed models successfully added fluorescence to accurately present tumor lesions. The combination of fluorescent lesions helps surgeons to familiarize themselves with the surgical scene, accurately identify and locate lesions, and improve the proficiency of minimally invasive surgery. Moreover, the addition of fluorescence to liver lesions may further improve accuracy of minimally invasive resection in complex liver cancer.

Although 3D printing technology itself may not be new, the integration of fluorescent assembly with reusability and its application in the production of liver cancer models represents an innovative consolidation. This consolidation may bring unprecedented convenience and efficiency improvements over previous technologies [21, 22, 26, 27]. The practical application value of this model should be emphasized. It can be utilized for both specific treatment processes and the production of teaching aids, with the specific application depending on actual needs and scenarios. If applied in specific treatment processes, this method indeed has the potential to optimize treatment outcomes. The indicators for optimization may include the accuracy of surgical operations, surgical duration, and improvements in patient recovery. These indicators reflect surgical outcomes and patients’ quality of life, thereby assessing whether the treatment has been optimized. If used for creating teaching aids, this method can similarly enhance the effectiveness of teaching. The indicators for optimization may encompass students’ academic performance, improvements in learning interest and motivation, and the mastery of practical operational skills. These indicators measure students’ understanding and mastery of knowledge, as well as whether the teaching effectiveness has been optimized.

It’s worthwhile to expect that the use of such reusable 3D-printed models also optimizes time and energy. In treatment processes, it assists doctors in more accurately planning surgeries, reducing unnecessary surgical time, and consequently lowering surgical risks and patient recovery time. In teaching, it provides students with an intuitive and vivid learning tool, aiding them in quicker comprehension and mastery of relevant knowledge, thus improving efficiency. Furthermore, when compared with other existing technologies or methods [21, 22, 26, 27], the advantages of this 3D-printed model should be highlighted. For instance, it may offer higher precision, shorter production cycles, or lower costs. It may also drive further application and development of 3D printing technology in the medical and educational fields, or provide new ideas and methods for related research areas. However, more experiments need to be conducted in the future to further explore and optimize the application of this 3D-printed model with more solid data.

Additionally, our model development and design embody a concept of repeatable training. Specifically, the tumor tissue that needs to be resected is designed as hydrogel, while the remaining tissue structures are designed as silicone. By repeatedly replacing the hydrogel models, repeated training can be achieved. This concept can also be applied to other organs, such as the lungs, uterus, kidneys, spleen, gastrointestinal tract, and more. The tumors at segment 4a and segment 8 that we selected represent complex liver tumors in corresponding regions, and tumors in other parts of these regions can also be designed in corresponding hydrogel models.

While the current study offers valuable insights, it is crucial to acknowledge its limitations. First, as this study is in the initial stage of model development, there are not sufficient cases for statistical validation, which constitutes a limitation. It is worth acknowledging that future research should include rigorous statistical methods to enhance the robustness of the findings. Second, although this study demonstrates the model development process, it lacks validation of reliability and validity. In the future, with the accumulation of more cases, we can design questionnaires and conduct more expert evaluations to verify the effectiveness and reliability of the model. Third, although the value provided in this paper is limited, presenting only individual cases of the model and lacking scientific exploratory statistical analysis with more data, this study offers an interesting model development, embodying an engineering design concept and a technical implementation scheme. Fourth, future research can conduct better-designed comparisons with VR to determine whether our model truly further enhances training efficiency and reduces costs.

## 5. Conclusion and outlook

The reusable fluorescent assembled 3D-printed models may mimic minimally invasive resection of complex liver cancer, demonstrating potential value in simulated surgery. The workflow allows for the establishment of repeated simulated surgery for different complex liver cancers based on the location of each patient’s lesions. Our models may help surgeons improve their proficiency in key surgical steps and provide valuable hands-on opportunities for young surgeons. However, our model development is still in its preliminary stage and requires more cases to verify its practicality. Future research can attempt to simulate training with more cases involving tumors in different locations.
